# The role of progranulin in diabetes and kidney disease

**DOI:** 10.1186/s13098-015-0112-6

**Published:** 2015-12-21

**Authors:** Bruna Bellincanta Nicoletto, Luis Henrique Canani

**Affiliations:** Post Graduation Medical Sciences Program: Endocrinology, School of Medicine, Universidade Federal do Rio Grande do Sul (UFRGS), 2400 Ramiro Barcelos Street, 2º floor, Porto Alegre, Rio Grande do Sul 90035-003 Brazil; Division of Endocrinology, Hospital de Clínicas de Porto Alegre, 2350 Ramiro Barcelos Street, Building 12, 4° floor, Porto Alegre, 90035-903 Brazil

**Keywords:** Progranulin, Adipokine, Obesity, Diabetes, Kidney disease, Inflammation

## Abstract

Progranulin (PGRN) is a cysteine rich secreted protein, expressed in epithelial cells, immune cells, neurons, and adipocytes. It was first identified for its growth factor-like properties, being involved in early embryogenesis and tissue remodeling, acting as an anti-inflammatory molecule. In the central nervous system, PGRN has neurotrophic and neuroprotective actions. There is also evidence of PGRN effects on cancer, contributing to tumor proliferation, invasion and cell survival. Recently, PGRN was recognized as an adipokine related to obesity and insulin resistance, revealing its metabolic function and pro-inflammatory properties. In obesity and type 2 diabetes mellitus, PGRN levels are increased. In renal disease, there is a relevant association, however, it is not known if it could contribute to kidney damage or if it is only a route of PGRN elimination. PGRN is an emerging molecule which demands studies in different fields. Possibly, it plays distinct functions in different tissues/cells and metabolic conditions. Here, we discuss potential mechanisms and recent data of PGRN pro-inflammatory actions, regarding obesity, insulin resistance, type 2 diabetes mellitus and kidney disease.

## Background

Progranulin (PGRN) is a 68–88 kDa cysteine rich secreted protein, also known as granulin-epithelin precursor, proepithelin or PC-cell derived growth factor [1, 2]. It is encoded by *GRN* (PGRN gene) and expressed in many cell types, including epithelial cells, immune cells, neurons, and adipocytes [3]. In kidneys, PGRN is expressed by renotubular epithelia of mouse embryo [4]. In healthy adult rodents, PGRN continues to be expressed in the kidney, strongly in the transitional epithelium of the ureter; but weakly in the proximal and distal convoluted tubules of the cortex and collecting ducts of the medulla [5]. In humans, the PGRN expression in kidneys remains unknown.

The first evidence of the protein was found during guinea pig spermatogenesis, when an acrosomal glycoprotein, named acrogranin, was detected [6]; and later identified as the guinea pig equivalent of PGRN [7]. PGRN has growth factor–like properties, being involved in early embryogenesis [4], wound repair and tissue remodeling [8]. It regulates cell division, survival, and migration, mainly via extracellular regulated kinase (ERK) and phosphatidylinositol 3-kinase (PI3K) pathways [8]. The growth factor–like properties of PGRN could be involved in physiology of tissue repair or in diseases such as cancer [1]. By the same pathways (ERK and PI3K), PGRN contributes to tumor proliferation, invasion and cell survival [9–11]. This molecule has previously been linked to many cancer types, as breast [12], ovarian [13], cervical [14], gastrointestinal [15] and kidney cancers [16].

Progranulin is secreted in an intact form and can be cleaved into granulins by proteases [2, 3]. Granulins are small proteins of approximately 6 kDa characterized by a conserved motif of 12 cysteines and play a role in the extracellular regulation of cell function and growth [8]. It has been suggested that the full length form of the protein (PGRN) has anti-inflammatory action, while released granulins have the opposite effect, increasing the production of proinflammatory cytokines, as interleukin 8 [1]. The intact PGRN is reported bind to tumor necrosis factor receptor (TNFR), inhibiting the binding of tumor necrosis factor-α (TNFα) and its proinflammatory signaling [17, 18]. In mouse models of arthritis, PGRN prevents inflammation [17]. In humans, increased serum levels of PGRN are observed in rheumatoid arthritis, but its relation to the pathogenesis of the disease remains unclear [19]. Elevated PGRN levels are observed in the skin of psoriasis patients [20, 21], mice dermatitis model [20, 21] and wounds [1]. Some authors suggest that PGRN has the effect of attenuating inflammation in these conditions, acting as an anti-inflammatory molecule [20, 21].

In central nervous system, PGRN has neurotrophic and neuroprotective actions [22]. It is involved in neurite outgrowth and possibly plays a role in plasticity and remodeling in the adult brain [23]. In addition, PGRN protects neurons from premature death [8] and acts in the response to stress [23] and neuroinflammation [22]. PGRN deficiency is associated with neurodegeneration and frontotemporal dementia (FTD), mainly due to mutation in *GRN* [24]. However, PGRN expression is upregulated in microglia in neurodegenerative disease [25] as FTD, especially in brain areas with a substantial pathology [26]. It is unclear if it represents a result of microglia response to injury or an active contribution to the disease progression [22].

After acute ischemia–reperfusion injury, lower PGRN levels are observed in mice brain [27] and kidney [28]; and treatment with recombinant PGRN could attenuate inflammation in this condition [27, 28]. PGRN also seems to protect against acute focal cerebral ischemia in rats by attenuation of blood–brain barrier disruption, neuroinflammation suppression, and neuroprotection [29].

Despite the reported anti-inflammatory properties of PGRN in some conditions, it seems to be a more complex molecule, revealing an opposite metabolic function. In the periphery, the intact form of PGRN has been associated with proinflammatory effects, since PGRN was recently recognized as an adipokine related to obesity and insulin resistance [3, 30]. Nothing further is known about the relationship of PGRN with its proteolytic granulins in obesity and insulin resistance.

## Review

### Adipose tissue, obesity and PGRN

Since the discovery of leptin in 1994, adipose tissue has been recognized as an endocrine organ, with its secreted adipokines playing many functions in the body [31]. Leptin acts on energy metabolism, regulating appetite and food intake, as an anorexigenic hormone [32]. In obesity, its levels are increased; however, leptin resistance is observed, impairing leptin functions [33]. A similar biological process is suggested for PGRN [34]. There is evidence that the administration of PGRN in the mice hypothalamus significantly suppresses fasting-induced feeding and body weight gain in a dose-dependent manner, possibly through hypothalamic neuropeptide Y and the melanocortin system [34]. However, in obesity, a resistance to the anorexigenic effects of PGRN may contribute to increased food intake [34].

In obesity, PGRN levels are increased [30, 35, 36]. Both experimental studies [35] and those performed in humans [30, 36] have reported the relationship between PGRN and adiposity. In *ob/ob* mice, a well-characterized obese and insulin resistance model, there are elevated PGRN serum levels and upregulation of *Grn* in white adipose tissue [35]. In humans, previous studies report a positive correlation between body mass index (BMI) and PGRN serum levels [30, 36, 37].

Progranulin is positively correlated to body fat percentage and waist circumference [36–38]. Fat distribution is related to PGRN levels, revealing higher PGRN serum concentration in subjects with visceral obesity [36]. It is known that visceral fat and its secreted adipokines and immune cell-derived cytokines are involved in chronic inflammation [31]. PGRN seems to play a role on this process, due its chemotactic activity, recruiting monocytes into adipose tissue as well as monocyte chemoattractant protein-1 (MCP-1) [36]. Furthermore, there is evidence that *Grn* deficient mice had significantly less infiltration of macrophages in adipose tissue [35]. Taken together, these findings suggest a proinflammatory effect of PGRN. This is supported by human studies that found a positive correlation between PGRN and C-reactive protein (CRP) [36, 39] and interleukin 6 (IL-6) [30, 39].

The effects of PGRN in obesity and inflammation have also revealed its influence on insulin resistance. A positive correlation between PGRN levels and HOMA-IR index (*Homeostasis Model Assessment for insulin resistance*) has been reported [30, 37]. In morbid obesity, patients with insulin resistance have elevated PGRN serum concentration [40].

**Fig. 1:**
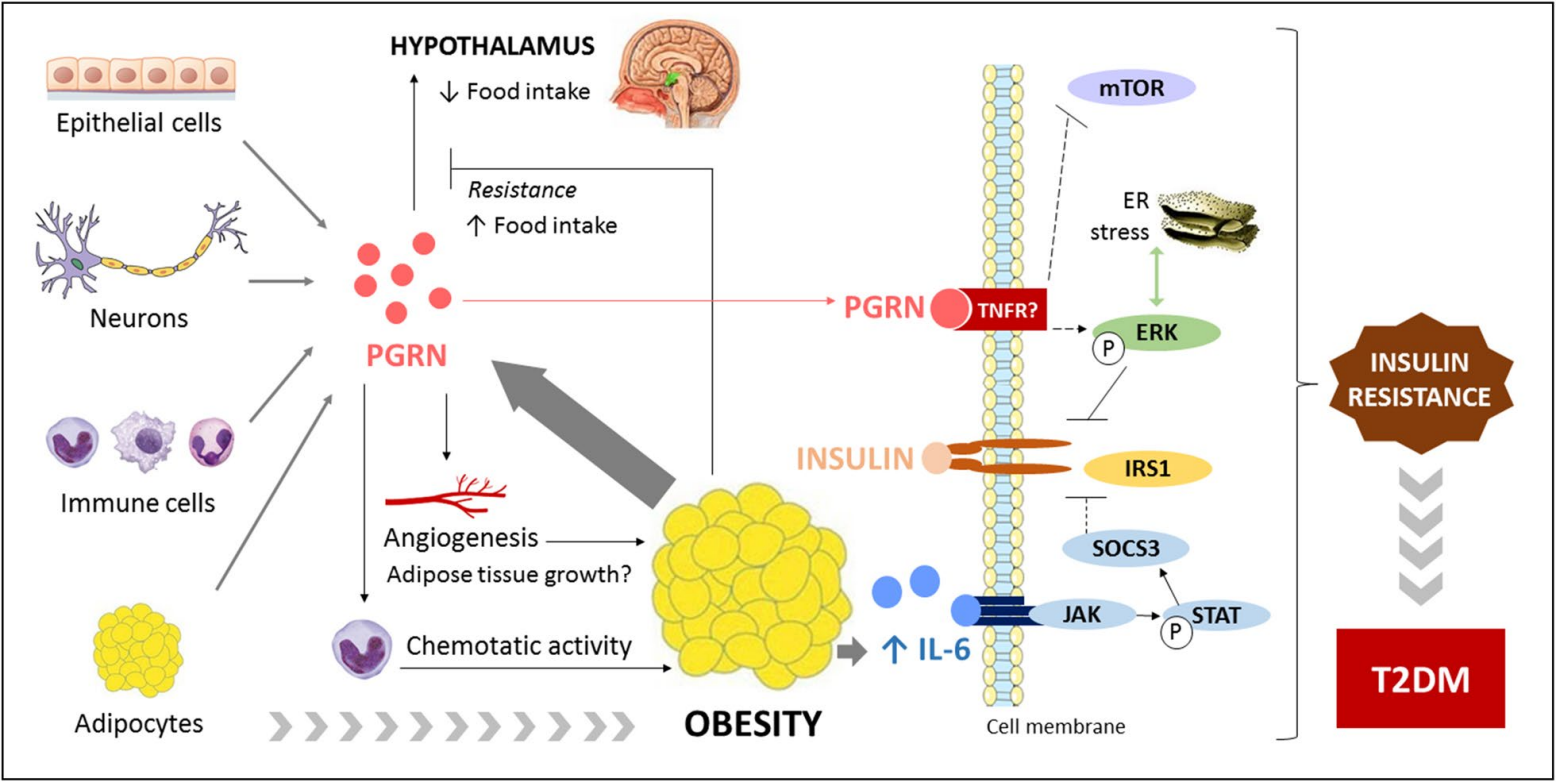
Association of PGRN with obesity, insulin resistance and type 2 diabetes mellitus. *PGRN* progranulin, *IL-6* interleukin-6, *TNFR* tumor necrosis factor receptor, *mTOR* mammalian target of rapamycin, *ERK* extracellular regulated kinase, *IRS-1* insulin receptor substrate-1, *SOCS3* cytokine signaling-3, *T2DM* type 2 diabetes mellitus

Experimental studies reported that PGRN promotes IL-6 expression in adipose cells, and its elevation enhances cytokine signaling-3 (SOCS3) expression via activation of JAK-STAT signaling. This mechanism can inhibit tyrosine phosphorylation of insulin receptor substrate-1 (IRS-1), leading to insulin resistance [35]. Additional evidence reports that *Grn* deficient mice fed with high fat diet presented improved insulin sensitivity. Moreover, body weight, fat mass and size of adipocytes were lower in *Grn* deficient mice compared to the wild-type mice receiving a standard diet [35]. These findings suggest a relevant association of PGRN with obesity and insulin resistance [35], as summarized in Fig. 1.

Recently, experimental studies reported that PGRN increases autophagic activity and triggers endoplasmatic reticulum (ER) stress in cultured human adipocytes, impacting on insulin signaling [37]. First, in multiple insulin-resistant cellular models there were increased levels of PGRN and autophagic imbalance. In PGRN deficient adipocytes, decreased markers of autophagy were observed. Moreover, adipocytes treated with PGRN and then stimulated with insulin revealed diminished IRS-1 and Akt phosphorylation and increased autophagic disorder. Mechanisms suggested involve ERK and mammalian target of rapamycin (mTOR) pathways [37]. ERK activation impairs IRS-1 activity and is associated with autophagy and ER stress-induced insulin resistance [41]. Likewise, inhibition of mTOR reduces insulin action and promotes autophagic disorders in adipocytes [42]. Possibly, PGRN effects are mediated by ERK activation and impaired mTOR phosphorylation [37] (Fig. 1). Moreover, there is evidence that PGRN could exert a causative role in hepatic insulin resistance, as observed in mice treated with PGRN for 21 days. Animals presented impaired glucose and insulin tolerance, and hepatic autophagy imbalance [43].

Regulation of PGRN on autophagy disorders and insulin resistance seems to be partially mediated through TNFR-1 via NF-kB signaling [37, 43]. Previous studies revealed that PGRN binds to TNFR, impairing the TNFα/TNFR interaction and suppressing chronic inflammation in mouse models of arthritis [17]. Although one study failed to demonstrate the binding of PGRN to TNFR [44], a recent publication reinforces this interaction [45]. Further evidence is required to elucidate the effects of PGRN binding to TNFR in different tissues, but it is possible that PGRN has dual roles in inflammation, exhibiting pro- and anti-inflammatory proprieties.

Some authors suggest that PGRN could be involved in the growth of adipose tissue [3]. It is known that the expansion of fat mass during obesity is followed by angiogenesis [46]; and PGRN has previously been linked to vessel formation [47]. Therefore, this adipokine could also contribute to increase adiposity (Fig. 1). However, this hypotheses needs to be confirmed by further studies.

Regarding adipogenesis, the role of PGRN is not fully understood. Its anti-adipogenic effects were reported in a previous study using porcine preadipocytes [48]. PGRN promoted ERK activation that phosphorylate peroxisome proliferator-activated receptor-gamma (PPARg) at the serine 112 site, impairing its function on adipocyte differentiation [48]. The effect of PGRN on ER stress also suppressed adipogenesis in cultured human adipose cells [37]. Moreover, Matsubara et al. [35] found that PGRN expression decreased with differentiation of 3T3-L1 adipose cells assessed by pre-adipocyte factor-1, PPARg, and fatty acid binding protein. On the other hand, a positive significant correlation between circulating PPARg and PGRN was observed in obese subjects [49].

### Type 2 diabetes mellitus and PGRN

From 1988 to 2010, the total number of persons with diabetes increased by almost 75 % [50]. In 2012, there were an estimated 371 million adults living with diabetes, with 4.8 million deaths attributable to this disease [51].

Type 2 diabetes mellitus (T2DM) is characterized by a resistance to insulin action and inadequate compensatory insulin secretory response, leading to hyperglycemia [52]. Obesity and visceral fat are probably the main risk factors for T2DM [53] and are involved in its pathophysiology as well as inflammation and insulin resistance [31]. Genetic and T2DM family history are widely studied and several genes have been associated with T2DM development [54, 55].

The PGRN gene is located in the chromosome region 17q21.32. The long arm of chromosome 17 was previously linked to visceral adipose tissue, waist circumference and BMI in Hispanic families in the Insulin Resistance Atherosclerosis Family Study [56]. Moreover, linkage signals of fasting glucose was found with 17q in young European sib-pairs, suggesting an association with T2DM [57]. Other important genes are located in this chromosome region, as SOCS3 [58] and genes involved in food intake [59].

Recently, new markers have been studied in the pathogenesis of diabetes, involving many adipokines [60, 61], such as PGRN [30, 36, 38]. There is evidence that PGRN levels are increased in T2DM when compared to non-diabetic subjects [30, 36, 38, 62]. PGRN is closely related to glucose metabolism. There is a positive correlation between PGRN and A1C, fasting plasma glucose and 2 h post-challenge plasma glucose [30, 36, 37]. Elevated PGRN concentrations are also observed in impaired glucose tolerance subjects, revealing its role in prediabetic states [38]. Moreover, a recent study evaluating T2DM patients reports that obese subjects present higher levels of PGRN [30].

The association of PGRN with T2DM is mainly explained by its role in adipose tissue and insulin resistance. PGRN promotes IL-6 expression, impairing insulin signaling [35]. Moreover, it is a chemoattractant protein that recruits monocytes into adipose tissue, promoting inflammatory response with increased cytokines levels [36] (Fig. 1).

Decrease in circulating PGRN levels can be obtained with long term diet intervention [63] and exercise training [36]. A recent study evaluated the change in PGRN levels after 24 months of dietary intervention, and showed that the decrease was sustained throughout this period, irrespective of weight stabilization or partial weight regain [63]. Another study identified a significantly decrease of ~20 % in PGRN serum concentration after a 4-week training program, only in T2DM patients [36].

Other metabolic disorders associated with T2DM have also been linked to PGRN. A positive correlation observed between total cholesterol [36], triglycerides [30, 37] and PGRN suggests a role in dyslipidemia. Patients with metabolic syndrome present higher serum PGRN concentration [37, 64] and the number of metabolic syndrome components have a significant positive correlation with PGRN levels [39]. Elevation of PGRN expression in omental adipose tissue is also observed in patients with metabolic syndrome, indicating a potential contribution of adiposity to increased PGRN serum levels [37]. Moreover, patients with metabolic syndrome also present increased autophagic activity and ER stress in adipose tissue [37]. Finally, the effects of PGRN in obesity, insulin resistance and inflammation contribute to its association with atherosclerosis [39].

T2DM is associated with poor outcomes, characterized by macro and microvascular complications. Intensity and duration of hyperglycemia exposure leads to vascular and nervous damage, resulting in organ dysfunction, such as kidney, eyes, nerves, heart and blood vessels [52, 65].

### Kidney disease and PGRN

Chronic kidney disease (CKD) is defined as a reduced glomerular filtration rate (GFR), increased urinary albumin excretion (UAE), or both. The incidence and prevalence of CKD differ substantially across countries; however, the estimated worldwide prevalence is around 8–16 % [66].

Although hypertension, glomerulonephritis and other comorbidities can lead to development of renal dysfunction, diabetes is the main cause of CKD [66]. In Brazil, it is the primary kidney disease in most patients starting dialysis [67]. The prevalence of diabetic kidney disease (DKD) increases over the years after the T2DM diagnosis, affecting about 25 % of patients with 10 years of the pathology [68]. Hyperglycemia associated with hypertension can lead to glomerulus injury [69, 70]. Tissue inflammation promotes kidney fibrosis, leading to protein clearance, such as albuminuria [71]. Although increased UAE are common in DKD, some patients with T2DM present reduced GFR, even in the absence of albuminuria [69, 70, 72].

Chronic kidney disease complications include increased all-cause and cardiovascular mortality, kidney-disease progression, acute kidney injury, anaemia, mineral and bone disorders, fractures and cognitive decline [66]. When associated with diabetes, an increased risk of mortality is observed [67, 73].

Recently, PGRN was described as an adipokine dependent of renal function [74]. Five hundred thirty-two patients with stages 1–5 of CKD (according to the National Kidney Foundation classification) had their PGRN serum levels evaluated. Even after adjustment for age, sex and BMI, PGRN remained significantly different between the five subgroups of CKD, being higher in stage 5. Moreover, estimated GFR was identified as an independent predictor of PGRN circulating levels. These findings suggest that renal filtration is an important route of PGRN elimination [74]. However, the authors did not find a significant correlation between serum and urinary PGRN in a subgroup of 145 patients, which limits their conclusions. There is also an hypothesis that PGRN might contribute to the proinflammatory state frequently observed in renal disease [74].

Progranulin also seems to be involved in DKD. In a recent study evaluating eighty-four T2DM patients, increased PGRN serum levels were described in macroalbuminuric subjects [75]. The study also evaluated the presence of proliferative diabetic retinopathy and observed higher levels of PGRN in this group of patients, suggesting PGRN as a marker for diabetic microangiopathy and its severity [75].

The association of urinary PGRN levels and renal damage was investigated in seventy-four patients with type 1 diabetes mellitus (T1DM) [76]. Subjects were evaluated at baseline, when urine was collected, and after 6 years, when albuminuria and early renal function decline (ERFD, defined as a decline in cystatin C-based estimated GFR of ≥3.3 % per year) were assessed. Patients with both ERFD and albuminuria presented higher urinary PGRN levels at baseline than patients who maintained normal renal function and normoalbuminuria, when adjusted by age, diabetes duration, baseline albumin excretion rate, HbA1C, cystatin C and uric acid. Moreover, PGRN was significantly predictive of ERFD and albuminuria in patients with type 1 diabetes in multivariable logistic regression [76]. The study also investigated urinary levels of Tamms–Horsfall glycoprotein, clusterin and human α-1 acid glycoprotein; and concludes that a panel of these three proteins plus PGRN could be used to predict early signs of DKD [76]. Table 1 summarizes present data regarding PGRN and renal function.

**Table 1:** Studies characteristics regarding PGRN and renal function

Characteristic/reference	Xu et al. [75]	Richter et al. [74]	Schlatzer et al. [76]
Patients	84 patients with T2DM and 12 health persons	532 patients with stages 1–5 of CKD	74 patients with T1DM
PGRN material	Serum	Serum	Urine
Design	Cross-sectional study	Cross-sectional study	Longitudinal study Baseline: urine collection, PGRN dosage 3 and 6-year visit: assessment of MA and ERFD
Results regarding PGRN	PGRN serum levels are increased in T2DM patients with macroalbuminuria Positive correlation between serum PGRN and urinary albumin excretion rate Negative correlation between PGRN and eGFR	PGRN serum levels are different between groups of CKD stages ↑ PGRN levels at stage 5 of CKD CKD stage or eGFR are independently associated with PGRN serum levels	Lowest PGRN levels in patients who maintained normal renal function and normoalbuminuria (n = 35) Nonsignificant increase in patients with either ERFD (n = 15) or MA (n = 16) Significant increase in patients with both ERFD and MA (n = 8) Urinary PGRN was significantly predictive of ERFD and MA in patients with T1DM
Conclusion	PGRN might be considered as a marker for diabetic microangiopathy and its severity	Renal function assessed as eGFR is a strong, independent predictor of serum PGRN PGRN serum levels significantly increase with deteriorating renal function assessed as CKD stage	A panel of 4 proteins (PGRN, Tamms-Horsfall glycoprotein, clusterin and human α-1 acid glycoprotein) could be used to predict early signs of DKD

*CKD* chronic kidney disease, *T1DM* type 1 diabetes mellitus, *PGRN* progranulin, *ERFD* early renal function decline, *eGFR* estimated glomerular filtration rate, *MA* micro- or macroalbuminuria, *DKD* diabetic kidney disease

There is little evidence regarding the association of PGRN and DKD in T2DM patients. The proinflammatory effects of this adipokine could be involved in the pathway of renal damage, decreasing GFR and increasing albuminuria. When CKD is established, PGRN clearance is reduced and its effects could be potentiated. However, further studies are needed to elucidate this hypothesis.

In an acute condition, such as renal ischemia–reperfusion injury, an experimental study observed lower levels of PGRN in the mice kidney [28]. Moreover, *Grn* deficient mice presented a higher elevation of serum creatinine and blood urea nitrogen, more severe morphological injury and higher inflammatory response. Administration of recombinant PGRN in vitro could attenuate inflammation after renal ischemia–reperfusion injury at least in part associated with a nucleotide-binding oligomerization domain containing 2 (NOD2)-mediated immune response [28]. Therefore, PGRN plays a protective role and has an anti-inflammatory effect in the kidney after renal ischemia–reperfusion injury [28].

## Conclusions

Progranulin is an emerging molecule which demands studies in different fields. Previous data have identified PGRN as a pro- and anti-inflammatory protein. Possibly, it plays distinct functions in different tissues/cells and metabolic conditions, as reported in Table 2. It was previously demonstrated that expression of PGRN in intact skin is low, but in injured skin, it raises significantly [1]. In addition, PGRN exerts anorexigenic effect in lean state, but a resistance is observed in obesity, leading to increased food intake [34]. Moreover, in acute condition of ischemia–reperfusion injury, PGRN plays an anti-inflammatory effect [27–29], while in obesity (a chronic condition), it is associated with insulin resistance and inflammation [35].

**Table 2:** Metabolic conditions associated with pro- or anti-inflammatory effects of PGRN

Proinflammatory	Anti-inflammatory
Obesity Insulin resistance T2DM Dyslipidemia Metabolic syndrome	Wound repair Psoriasis Central nervous system Arthritis Acute ischemia–reperfusion injury

*T2DM* type 2 diabetes mellitus

It is not fully understood if PGRN is a cause or consequence of some conditions. PGRN could be involved in the pathogenesis of obesity and T2DM, and become a target for metabolic disorders prevention or treatment. In renal disease, it is not known if it could contribute to kidney damage or if it is only a route of PGRN elimination. In the last case, PGRN could be used as a marker of renal disease. Further studies are necessary to elucidate these questions and investigate the crosstalk between pro- and anti-inflammatory PGRN proprieties in different tissues and conditions, in order to clarify the action mechanisms of this potential molecule.

## Abbreviations

PGRN: progranulin
ERK: extracellular regulated kinase
PI3 K: phosphatidylinositol 3-kinase
FTD: frontotemporal dementia
TNFR: tumor necrosis factor receptor
TNFα: tumor necrosis factor-α
BMI: body mass index
MCP-1: monocyte chemoattractant protein-1
CRP: C-reactive protein
IL-6: interleukin 6
HOMA-IR: Homeostasis Model Assessment for insulin resistance
SOCS3: cytokine signaling-3
IRS-1: insulin receptor substrate-1
ER: endoplasmatic reticulum
mTOR: mammalian target of rapamycin
PPARg: peroxisome proliferator-activated receptor-gamma
T2DM: type 2 diabetes mellitus
CKD: chronic kidney disease
GFR: glomerular filtration rate
UAE: urinary albumin excretion
DKD: diabetic kidney disease
T1DM: type 1 diabetes mellitus
ERFD: early renal function decline
NOD2: nucleotide-binding oligomerization domain containing 2
